# Modified Bethesda system informing cytopathologic adequacy improves malignancy risk stratification in nodules considered benign or atypia(follicular lesion) of undetermined significance

**DOI:** 10.1038/s41598-018-31955-9

**Published:** 2018-09-10

**Authors:** You-Bin Lee, Ji-Ye Kim, Haeyon Cho, Soo Yeon Hahn, Jung Hee Shin, Seung-Eun Lee, Ji Eun Jun, Sun Wook Kim, Jae Hoon Chung, Tae Hyuk Kim, Young Lyun Oh

**Affiliations:** 1 Division of Endocrinology and Metabolism, Department of Medicine, Korea University Guro Hospital, Korea University College of Medicine, Seoul, Republic of Korea; 2 Division of Endocrinology and Metabolism, Department of Medicine, Samsung Medical Center, Sungkyunkwan University School of Medicine, Seoul, Republic of Korea; 3 Department of Pathology, Samsung Medical Center, Sungkyunkwan University School of Medicine, Seoul, Republic of Korea; 4 Department of Radiology, Samsung Medical Center, Sungkyunkwan University School of Medicine, Seoul, Republic of Korea; 5 Division of Endocrinology and Metabolism, Department of Medicine, Kyung Hee University School of Medicine, Kyung Hee University Hospital at Gangdong, Seoul, Republic of Korea

## Abstract

We modified the nondiagnostic/unsatisfactory category of the Bethesda system for reporting thyroid cytopathology to inform cytopathologic adequacy to better stratify the malignancy risk. Malignancy rates from 1,450 cytopathologic specimens not satisfying adequacy criteria from April 2011 to March 2016 were calculated based on sub-classification of the nondiagnostic/unsatisfactory category and sonographic patterns using matched surgical pathology. Rates were compared with those of 1,446 corresponding adequate specimens from July to December 2013. Upon resection, 63.2% of nondiagnostic, 36.7% of unsatisfactory + benign, 72.5% of unsatisfactory + atypia (follicular lesion) of undetermined significance, 98.1% of unsatisfactory + suspicious for malignancy, and 100.0% of unsatisfactory + malignant cases were confirmed to be malignant on surgical pathology. In nodules with inadequate specimens, those with high suspicion sonographic patterns had a malignancy rate (93.2%) higher than the others (45.5%) (*p* < 0.001). Nodules with unsatisfactory + benign specimens had a higher malignancy rate (36.7%) than satisfactory benign specimens (14.3%) (*p* = 0.020). For atypia (follicular lesion) of undetermined significance, the malignancy rate of inadequate specimens (72.5%) was higher than that of adequate specimens (51.3%) (*p* = 0.027). Sparse cellular samples with a few groups of benign follicular cells should not represent a benign lesion. There might be value in qualifying atypia (follicular lesion) of undetermined significance cases less than optimal.

## Introduction

Thyroid fine needle aspiration (FNA) is the standard tool for investigating and triaging thyroid nodules for surgery or clinical observation^1–3^. The Bethesda system for reporting thyroid cytopathology (TBSRTC) was set up by the 2007 National Cancer Institute meeting, which standardized the reporting of thyroid cytopathology^1,3,4^. Six diagnostic categories in this system are linked to certain ranges of malignancy risk and clinical management guidelines^1,4,5^. However, the nondiagnostic/unsatisfactory (ND/UNS) category among the six tiers of TBSRTC is a major limitation of thyroid FNA^2^.

Although TBSRTC suggests that ND/UNS results should be ideally limited to less than 10% of all thyroid FNA specimens^5^, the actual percentage ranged up to 23.6% according to a meta-analysis^3^. Factors that affect the rates of inadequate FNA include the experience of aspirators and cytopathologists, onsite adequacy assessment, and adequacy criteria^6^. However, inadequacy rates also depend on the characteristics of the nodule itself, such as size or radiological features^6^. Regarding the high percentage of ND/UNS cases, Renshaw pointed out that published studies were performed in academic centers or large reference centers where technically challenging cases are sent for aspiration^3,7^. In these nodules in which aspirations are technically difficult, it may be challenging to obtain an adequate sample even with repeat FNAs. Moreover, nodules with inadequate specimens have been managed in various ways in previous series^8^. Therefore, malignancy risk of inadequate FNAs, which are obtained in a relatively large proportion of patients, should be predicted using information like cytopathologic features and sonographic findings of the thyroid nodules. It is essential to establish proper and consistent guidelines for the management of thyroid nodules with inadequate aspirates.

In our center, we modified the ND/UNS category to suit the needs of clinicians, and we commented on the sample quality (less than optimal) with five TBSRTC categories (from ‘unsatisfactory + benign’ to ‘unsatisfactory + malignant’). We expected that these modifications would enhance quality control of aspirators by indicating the adequacy of specimens. We also tried to reduce the high non-diagnosable rates that occur frequently at tertiary referral centers and result in difficulties in clinical decisions. The main objective of this study was to determine the risk of malignancy (ROM) in thyroid nodules in the ND/UNS category. These samples were re-categorized according to our modified classification system, and their ROM was compared with that of corresponding adequate specimens. We also investigated the sonographic patterns of these nodules with inadequate FNAs, as this information is of clinical relevance for determining ROM^9^.

## Results

### Characteristics of the study population and included nodules

During the five-year study period, 1,450 thyroid aspirations from 1,423 patients were included (Supplementary Table S1). The mean age of the study population was 52.5 $\pm$ 11.9 years. Of the 1,423 individuals, 1,058 (74.3%) were females, and 365 (25.7%) were males. The median nodule size was 1.20 cm. Of 1,450 thyroid nodules, 213 (14.7%) were surgically resected.

### Malignancy rates in nodules with ND/UNS cytopathology

The maximum and minimum malignancy rates according to the six ND/UNS categories and sonographic patterns are presented in Table 1. Of the 213 excised nodules, 63.2% (12 of 19) of ND FNAs, 36.7% (22 of 60) of UNS + benign aspirates, 72.5% (50 of 69) of UNS + atypia (or follicular lesion) of undetermined significance (AUS/FLUS), 98.1% (53 of 54) of UNS + suspicious for malignancy (SM), and 100.0% (9 of 9) of UNS + malignant cytology cases were malignant on surgical pathology. However, the UNS + follicular neoplasm/suspicious for a follicular neoplasm (FN/SFN) category did not have enough cases to assess the ROM. Among the 146 malignant nodules confirmed by surgical pathology, 136 (93.2%) cases were papillary thyroid carcinomas, 8 (5.5%) cases were follicular thyroid carcinomas, and the rest two nodules included poorly differentiated carcinoma and medullary thyroid carcinoma, respectively (Supplementary Table S2).

**Table 1:** Malignancy rates for thyroid nodules that did not meet adequacy criteria and subsequent resection according to sonographic patterns and six nondiagnostic or unsatisfactory subcategories modified from the Bethesda System for Reporting Thyroid Cytopathology from April 2011 to March 2016.

FNA cytopathology	ATA nodule sonographic patterns	All FNAs (Number)	Surgically resected nodules (Number)	Malignant nodules confirmed by surgery (Number)	Malignancy rate	
					% maximum^*^	% minimum^†^
Nondiagnostic	High suspicion	30	9	8	88.9%	26.7%
	Others	201	10	4	40.0%	2.0%
	Overall	231	19	12	63.2%	5.2%
Unsatisfactory + Benign	High suspicion	76	10	7	70.0%	9.2%
	Others	836	50	15	30.0%	1.8%
	Overall	912	60	22	36.7%	2.4%
Unsatisfactory + AUS/FLUS	High suspicion	79	35	32	91.4%	40.5%
	Others	132	34	18	52.9%	13.6%
	Overall	211	69	50	72.5%	23.7%
Unsatisfactory + FN/SFN	High suspicion	0	0	0	NA	NA
	Others	3	2	0	0.0%	0.0%
	Overall	3	2	0	0.0%	0.0%
Unsatisfactory + Suspicious for malignancy	High suspicion	54	41	41	100.0%	75.9%
	Others	21	13	12	92.3%	57.1%
	Overall	75	54	53	98.1%	70.7%
Unsatisfactory + Malignant	High suspicion	17	8	8	100.0%	47.1%
	Others	1	1	1	100.0%	100.0%
	Overall	18	9	9	100.0%	50.0%
Total	High suspicion	256	103	96	93.2%	37.5%
	Others	1194	110	50	45.5%	4.2%
	Overall	1450	213	146	68.5%	10.1%

Abbreviations: FNA: fine-needle aspiration; ATA: American Thyroid Association; AUS/FLUS: atypia (or follicular lesion) of undetermined significance; FN/SFN: follicular neoplasm or suspicious for a follicular neoplasm; NA: not applicable.

^*^Percentage of cases calculated from total number of resected cases in each category (maximum malignancy rate).

^†^Percentage of cases calculated from total number of fine-needle aspirations in each category (minimum malignancy rate).

High suspicion sonographic patterns were associated with increased ROM in ND/UNS nodules. The maximum malignancy rate of ND/UNS nodules with a high suspicion ultrasound (US) pattern was 93.2%, while that of ND/UNS nodules without a high suspicion pattern was 45.5% (*p* for difference < 0.001). The difference in proportion was 47.7% [95% confidence interval (CI) 35.8–58.0%]. Moreover, the minimum malignancy rate of sonographically high suspicion ND/UNS nodules (37.5%) was 33.3% higher than that of ND/UNS nodules without a high suspicion US pattern (4.2%) (*p* for difference < 0.001). The 95% CI for the difference in proportion was 27.2–39.6%.

### Comparison of malignancy rates and diagnostic values in inadequate and adequate FNAs

Table 2 presents malignancy rates by TBSRTC category of the 1,446 specimens that met the adequacy criteria. They were compared to the malignancy rates of 1,219 inadequate specimens (Table 3). In AUS/FLUS, the maximum and minimum malignancy rates of inadequate FNAs were significantly higher than those of adequate specimens (*p* for difference 0.027 and 0.007, respectively). When the UNS + AUS/FLUS samples were compared with adequate AUS/FLUS specimens, the difference in maximum malignancy rate was 21.2% (95% CI 0.9–40.5%), while the difference in minimum malignancy rate was 11.1% (95% CI 2.8–19.0%). Conversely, the maximum and minimum malignancy rates for SM or malignant samples did not show a significant difference according to satisfaction of adequacy criteria.

**Table 2:** Malignancy rates for thyroid nodules that met adequacy criteria and subsequent resection according to cytopathologic categories based on the Bethesda System for Reporting Thyroid Cytopathology from July to December 2013.

FNA cytopathology	All FNAs (Number)	Surgically resected nodules (Number)	Malignant nodules confirmed by surgery (Number)	Malignancy rate	
				% maximum^*^	% minimum^†^
Benign	857	35	5	14.3%	0.6%
AUS/FLUS	159	39	20	51.3%	12.6%
FN/SFN	20	9	3	33.3%	15.0%
Suspicious for malignancy	62	40	39	97.5%	62.9%
Malignant	348	222	222	100.0%	63.8%
Total	1446	345	289	83.8%	20.0%

Abbreviations: FNA: fine-needle aspiration; AUS/FLUS: atypia (or follicular lesion) of undetermined significance; FN/SFN: follicular neoplasm or suspicious for a follicular neoplasm.

^*^Percentage of cases calculated from total number of resected cases in each category (maximum malignancy rate).

^†^Percentage of cases calculated from total number of fine-needle aspirations in each category (minimum malignancy rate).

**Table 3:** Comparison of malignancy rates for inadequate and adequate fine needle aspirations.

FNA results	Maximum malignancy rate			Minimum malignancy rate		
	Inadequate FNAs^*^	Adequate FNAs^†^	*p*-value	Inadequate FNAs^*^	Adequate FNAs^†^	*p*-value
Benign	22/60 (36.7%)	5/35 (14.3%)	**0.020** ^‡^	22/912 (2.4%)	5/857 (0.6%)	**0.002** ^‡^
AUS/FLUS	50/69 (72.5%)	20/39 (51.3%)	**0.027** ^‡^	50/211 (23.7%)	20/159 (12.6%)	**0.007** ^‡^
FN/SFN	0/2 (0.0%)	3/9 (33.3%)	1.000^§^	0/3 (0.0%)	3/20 (15.0%)	1.000^§^
Suspicious for malignancy	53/54 (98.1%)	39/40 (97.5%)	1.000^§^	53/75 (70.7%)	39/62 (62.9%)	0.336^‡^
Malignant	9/9 (100.0%)	222/222 (100.0%)	NA	9/18 (50.0%)	222/348 (63.8%)	0.237^‡^
Total	134/194 (69.1%)	289/345 (83.8%)	**<0.001** ^‡^	134/1219 (11.0%)	289/1446 (20.0%)	**<0.001** ^‡^

Abbreviations: FNA: fine-needle aspiration; AUS/FLUS: atypia (or follicular lesion) of undetermined significance; FN/SFN: follicular neoplasm or suspicious for a follicular neoplasm; NA: not applicable.

^*^Cases were collected from April 2011 to March 2016.

^†^Cases were collected from July to December 2013.

^‡^Pearson’s Chi-square test was applied for comparison.

^§^Fisher’s exact test was performed for analysis.

The maximum and minimum malignancy rates of the UNS + benign category were significantly higher than those of benign FNAs meeting adequacy criteria (*p* for difference 0.020 and 0.002, respectively) (Table 3). The difference in proportion was 22.4% (95% CI 2.4–38.8%) for maximum malignancy rate and 1.8% (95% CI 0.6–3.1%) for minimum malignancy rate. Consistent with these results, the sensitivity and NPV of inadequate FNAs were significantly lower than those of adequate specimens (Table 4). Although the specificity was numerically higher for inadequate specimens compared to adequate ones, it was not statistically significant.

**Table 4:** Comparison of main diagnostic values for inadequate and adequate fine needle aspirations.

Diagnostic indicator	Inadequate FNAs^||^	Adequate FNAs^¶^	*p*-value
Sensitivity (malignant, suspicious for malignancy, FN/SFN, and AUS/FLUS)^*,†^	112/134 (83.6%)	284/289 (98.3%)	**<0.001**
Sensitivity (malignant, suspicious for malignancy, and FN/SFN)^‡,§^	62/84 (73.8%)	264/269 (98.1%)	**<0.001**
Specificity^*^	38/60 (63.3%)	30/56 (53.6%)	0.288
Specificity (excluding AUS/FLUS) ^‡^	38/41 (92.7%)	30/37 (81.1%)	0.128
PPV for malignant, suspicious for malignancy, FN/SFN, and AUS/FLUS^†^	112/134 (83.6%)	284/310 (91.6%)	**0.013**
PPV for malignant, suspicious for malignancy, and FN/SFN^§^	62/65 (95.4%)	264/271 (97.4%)	0.387
NPV for benign cytology	38/60 (63.3%)	30/35 (85.7%)	**0.020**
Diagnostic accuracy (malignant, suspicious for malignancy, FN/SFN, AUS/FLUS and benign)^*^	150/194 (77.3%)	314/345 (91.0%)	**<0.001**
Diagnostic accuracy (malignant, suspicious for malignancy, FN/SFN, and benign)^‡^	100/125 (80.0%)	294/306 (96.1%)	**<0.001**

Abbreviations: FNA: fine-needle aspiration; AUS/FLUS: atypia (or follicular lesion) of undetermined significance; FN/SFN: follicular neoplasm or suspicious for a follicular neoplasm; PPV: positive predictive value; NPV: negative predictive value.

^*^The non-diagnostic category was excluded.

^†^AUS/FLUS, FN/SFN, suspicious for malignancy, and malignant nodules with malignant surgical pathology were considered true-positives.

^‡^Cases interpreted as non-diagnostic or AUS/FLUS were excluded.

^§^FN/SFN, suspicious for malignancy, and malignant cases confirmed to be malignant were considered true-positives.

^||^Cases were collected from April 2011 to March 2016.

^¶^Cases were collected from July to December 2013.

### Malignancy rates in nodules with ND cytopathology according to its characteristics

Specimens in the ND category were sub-classified by the factors that led to ND categorization (Table 5). Insufficient cellularity accounted for most cases. Malignancy rates were individually calculated according to the ND characteristics (Table 5). Although there were not enough cases to ensure adequate evaluation, except in the ‘insufficient cellularity’ group, specimens in the ‘mainly calcified material only’ group showed a relatively high malignancy rate of minimum 16.7%.

**Table 5:** Malignancy rates for thyroid nodules with nondiagnostic fine needle aspirations according to characteristics of nondiagnostic cytopathology from April 2011 to March 2016.

Nondiagnostic cytology	All FNAs (Number)	Surgically resected nodules (Number)	Malignant nodules confirmed by surgery (Number)	Malignancy rate	
				% maximum^*^	% minimum^†^
Insufficient cellularity	189	17	10	58.8%	5.3%
Cystic fluid only	34^‡^	1	1	100.0%	2.9%
Obscuring blood, blood only	28^§^	3	2	66.7%	7.1%
Drying artifact	1^‡^	0	0	NA	0.0%
Mainly calcified material only	6^||^	1	1	100.0%	16.7%
Total	231	19	12	63.2%	5.2%

Abbreviations: FNA: fine-needle aspiration.

^*^Percentage of cases calculated from total number of resected cases in each category (maximum malignancy rate).

^†^Percentage of cases calculated from total number of fine-needle aspirations in each category (minimum malignancy rate).

^‡^One of these cases was also included in the subcategory of insufficient cellularity.

^§^Nineteen of these cases were also included in the subcategory of insufficient cellularity.

^||^All six of these cases were also included in the subcategory of insufficient cellularity.

## Discussion

We screened 16,321 thyroid FNAs that had been assessed for adequacy and seen over a five-year period at a tertiary referral center. We identified 1,450 thyroid FNAs that did not meet adequacy criteria and compared their ROMs with those of a historical control group of adequate FNAs. In reporting cytopathologic inadequacy along with TBSRTC category, we caution clinicians of the specimens’ limitations while providing the best information possible from the given specimen to enable further assessment with the help of complementary modalities, such as ultrasonographic findings. Importantly, this study demonstrated that differentiating inadequate FNAs does result in significantly different malignancy predictions, specifically in the benign and AUS/FLUS categories. These results imply that insufficient specimen quantities may under-represent rather than over-represent atypia.

According to TBSRTC, the presence of any atypia is one exception to the numerical adequacy criteria requirements^4,5^. FNAs with atypia are, by definition, considered satisfactory for evaluation^4,5^. In contrast, we sub-classified atypia-bearing specimens that did not meet adequacy criteria into UNS + AUS/FLUS, UNS + FN/SFN, UNS + SM, and UNS + malignant categories, and distinguished them from the adequate cases with atypia. In this study, the malignancy rates did not change with satisfaction of adequacy criteria in malignant or SM specimens. Therefore, meeting the adequacy criteria may not be necessary in these categories, as recommended by TBSRTC. However, in the AUS/FLUS category, malignancy rates of inadequate specimens were significantly higher than those of specimens meeting adequacy criteria. This suggests that adequacy of samples may be important to assess the ROM in AUS/FLUS, unlike in the malignant or SM category. Therefore, in AUS/FLUS samples, it may be useful to indicate specimen adequacy in cytopathologic reports. Recently, personalized approaches utilizing all available information including clinical, radiologic, and cytopathologic data, have been recommended to manage AUS/FLUS lesions^10^. Modifying TBSRTC to state satisfaction of adequacy criteria in AUS/FLUS specimens may provide useful information for individualized assessment. Moreover, AUS/FLUS results in inadequate samples may need to be managed with more prudence because of the substantially higher ROM. In particular, if AUS/FLUS nodules with inadequate specimens are accompanied by high suspicion patterns on sonography, they may need to be referred directly for surgery rather than for repeat FNA since the ROM in this group was at least 40.5% in this study.

Recent studies have suggested that the required number of follicular cells for adequate thyroid FNAs can be lowered without significantly affecting sensitivity^6,11^ and/or ROM^11^. However, in one of these reports^6^, only FNAs examined with ThipPrep were included, and aspirations with conventional smears were not considered. Another study by Renshaw^11^ was performed with 170 surgically-matched ND/UNS FNAs including only benign appearing follicular cells and those without Hürthle cells or atypia. Malignancy risk and test performances including sensitivity and specificity in these samples were compared by lowering the threshold of adequacy. Therefore, the ROM and diagnostic values were assessed only in ND/UNS specimens with benign appearing follicular cells, and they were not compared with completely adequate samples. In our study, the ROM in FNAs consistent with benign follicular nodules not fulfilling adequacy criteria was compared to that of benign specimens meeting adequacy criteria. The malignancy rates were significantly higher in inadequate specimens than in adequate ones. Also, in inadequate samples, the sensitivity was lower and false-negatives were higher compared to adequate specimens. These results suggest that meeting the adequacy criteria may be important to establish a benign FNA and to minimize false-negative results.

Ultrasonography plays an important role in stratifying the malignancy risk of thyroid nodules^12–14^. Malignancy risk-stratification systems have been developed to stratify the cancer risk of thyroid nodules based on sonographic features^13,15–17^. Recently, there has been an attempt to stratify the malignancy risk of thyroid nodules based on a scoring system that combines FNA cytology and ultrasound patterns^12^. However, the role of ultrasound in predicting cancer risk in nodules with ND/UNS FNAs only has not been sufficiently explored in previous reports. In this study, we demonstrated that ultrasonography plays an essential adjunct role in estimating malignancy risk, even in nodules in which FNA results did not meet the adequacy criteria.

In our study, the minimum malignancy rate was 5.2% in ND samples for which classification into a certain category was impossible. However, in ND samples with the subcategory of ‘mainly calcified material only’, the malignancy rate was estimated to be as high as at least 16.7%. Although calcification itself may result in inadequate nodule aspiration^1^, calcification within a solitary thyroid nodule has been reported to be associated with a high malignancy risk^18,19^. In a report by Khoo *et al*.^18^, 28 (75.7%) of 37 solitary nodules with intra-thyroidal calcification were carcinomas, and they recommended surgical resection in these lesions regardless of FNA results^18^. In our previous report^1^, two of three thyroid nodules with ND samples and malignant histology on resection showed calcification on preoperative sonography. Calcified materials in thyroid aspirations may be a useful finding that indicates an increased ROM. Referring to the presence of calcified material in reports of ND cytology may be helpful to estimate the malignancy risk. ND samples with only calcified material may need to be treated carefully to consider the possibility of cancer.

There are some limitations to our study. First, only three cases were classified as UNS + FN/SFN. Thus, the malignancy rate in this category could not be evaluated. Although this may be associated with a relatively lower prevalence of follicular thyroid cancer and a subsequently lower proportion of FN/SFN cases among thyroid FNAs in Korea compared to Western countries^1^, it may also be altered because FNA is unable to distinguish follicular lesions accurately^8^. Second, because our study was retrospective in design and the results of inadequate specimens were compared with those of adequate FNAs from a previous study, follow-up periods were not unified. Third, data were analyzed per nodule, not per participant, which may have led to an overestimation of our findings. However, when we performed the same analysis after excluding cases where two or more nodules were included from one patient, the results were not altered. Fourth, only 14.7% of thyroid nodules with inadequate FNAs were histologically confirmed by surgical pathology, suggesting that repeated FNA as recommended by TBSTRC or other clinical factors might have affected surgical triaging of these nodules with less than optimal specimens.

Taken together, the study results confirmed several tenets of TBSRTC approach to adequacy: 1) A sample should be considered ND/UNS if it is sparsely cellular, even if there are a few groups of benign follicular cells. 2) A case with any atypia should not be considered ND/UNS. The novel conclusion of our study is that there is likely value in qualifying AUS/FLUS cases that are less than optimal. The results need to be confirmed by other investigators before considering amendments to TBSRTC to inform cytopathologic adequacy.

## Methods

### Case selection and cytopathologic diagnosis

This study was conducted at a hospital-based tertiary referral center in Korea. In April 2011, our pathology department adopted TBSRTC to report thyroid FNA results according to the six-tier diagnostic scheme, and modified the criteria of the ND/UNS category of the Bethesda system. A total of 16,321 thyroid aspiration cases were retrieved from the medical records of the Pathology Department of the Samsung Medical Center in Seoul, from April 2011 to March 2016. Among these cases, only specimens that did not meet our adequacy criteria were retrospectively collected as follows. We excluded adequate FNAs satisfying numerical requirements (n = 14,836), which included at least six groups of well-visualized follicular cells and a minimum of ten cells per group^4,5^. According to TBSRTC recommendations, solid nodules with inflammation in which a specific diagnosis, such as lymphocytic thyroiditis or granulomatous thyroiditis, can be made are classified as benign, and numerical adequacy requirements do not need to be met in such nodules^4,5^. In our study, such specimens consistent with lymphocytic thyroiditis in the proper clinical context or granulomatous thyroiditis (n = 35) were excluded. We enrolled the remaining 1,450 thyroid aspirations from 1,423 patients and subdivided them as follows: “nondiagnostic (ND)” referred to samples that were completely acellular or extensively obscured with artifacts; “unsatisfactory (UNS) + benign” referred to samples that had some benign follicular cells organized into small macrofollicular fragments with portions of colloid but lacking the recommended quantity of follicular cells for a benign interpretation. The current TBSRTC recommends that whenever any significant atypia is present, the specimen is considered adequate regardless of whether adequacy criteria were satisfied^4,5^. However, according to our modified system, we stratified FNAs with atypia that had low cellularity or other obscuring features into “UNS + AUS/FLUS”, “UNS + FN/SFN”, “UNS + SM”, and “UNS + malignant”. These cytopathologic diagnoses were routinely made by the Pathology Department, and difficult cases were consulted on a single expert thyroid pathologist (YLO).

### Specimen preparation and review of ultrasound images

Either radiologists or endocrinologists performed thyroid FNAs with 22- or 23-gauge needles attached to 10-mL syringes under US guidance. Sonographic examinations were performed using a 5–12 MHz linear array transducer (iU22; Philips Medical Systems, Bothell, WA). For a nodule, an average of 2–4 passes was applied. Direct smears on glass slides were fixed immediately in 95% alcohol for Papanicolaou and hematoxylin and eosin staining.

All US images taken on the same day as FNAs were retrospectively reviewed. Sonographic features of nodules including composition (presence of solid components), echogenicity of the solid portion (marked hypoechoic, mild hypoechoic, isoechoic, or hyperechoic), orientation (parallel or non-parallel), and presence of microcalcification, extra-thyroidal extension or rim calcifications with small extrusive soft tissue components were analyzed. On the basis of these features, images of each nodule were interpreted according to recommendations of the 2015 revised American Thyroid Association (ATA) guidelines^13^ (Supplementary Table S3). Although the ATA guidelines^13^ suggest categorizing the sonographic patterns of thyroid nodules into five groups (high, intermediate, low, very low suspicion of malignancy, or benign), we applied a binary classification according to whether the US patterns were compatible with high suspicion nodules or not (high suspicion nodules versus others). High suspicion nodules were solid hypoechoic nodules or partially cystic nodules containing solid hypoechoic components with a minimum of one suspicious sonographic feature^13^. Suspicious US features included irregular margins, microcalcifications, non-parallel orientations, rim calcifications with small extrusive soft tissue components, and extra-thyroidal extension^13^.

### Histological follow-up

Cases included in the study were followed until February 2017 to determine whether they were surgically resected. FNA reports of resected nodules were matched to surgical pathology specimens. Surgical pathology subtypes included papillary thyroid carcinoma, follicular thyroid carcinoma, poorly differentiated thyroid carcinoma, medullary thyroid carcinoma, hyalinizing trabecular tumor, follicular adenoma, and benign follicular nodules (Supplementary Table S2), and no anaplastic thyroid carcinomas were observed. These categories were based on the World Health Organization (WHO) classification^20^. Among these subtypes, papillary thyroid carcinoma, follicular thyroid carcinoma, poorly differentiated thyroid carcinoma, and medullary thyroid carcinoma were considered as malignant histology. Malignancy rates were calculated according to six ND/UNS categories (ND, UNS + benign, UNS + AUS/FLUS, UNS + FN/SFN, UNS + SM, or UNS + malignant) and by binary classification of sonographic patterns. In the ND category, malignancy rates were also evaluated based on the ND subcategories, which were created according to the scenarios leading to ND result. They consisted of insufficient cellularity, cystic fluid only, obscuring blood, drying artifact, and mainly calcified material only.

Malignancy rates were estimated in two ways: maximum malignancy rate and the minimum malignancy rate^1,21^. The maximum malignancy rate was calculated only for resected nodules, and it was determined by dividing the number of surgically confirmed malignant nodules by the number of nodules chosen to undergo surgery. The minimum malignancy rate was estimated by dividing the number of surgically confirmed malignant nodules by the total number of nodules aspirated, whether or not they were resected. Noninvasive follicular thyroid neoplasm with papillary-like nuclear features (NIFTP) was not considered a malignancy^22^.

### Comparison with adequate aspirates

In our previous study^1^, FNAs for thyroid nodules performed from July to December 2013 were followed until February 2015 and retrospectively analyzed. The malignancy rate in each TBSRTC category was then calculated. To compare malignancy rates of inadequate specimens with those of adequate ones, we excluded FNAs that did not meet the adequacy criteria from that cohort and created a subgroup. Among a total of 1,925 FNAs reported according to TBSRTC, 1,446 thyroid FNAs from 1,359 patients were selected after excluding specimens consistent with lymphocytic thyroiditis in the proper clinical context or granulomatous thyroiditis, and other FNAs that did not meet adequacy requirements. Malignancy rates according to TBSRTC category were re-calculated only for adequate cases. The malignancy rates were presented with maximum and minimum values. A Pearson’s Chi-square test or Fisher’s exact test was performed to compare the maximum and minimum malignancy rates of inadequate specimens to those of adequate FNAs.

The diagnostic values of sensitivity, specificity, positive predictive value (PPV) and negative predictive value (NPV) for inadequate and adequate FNAs were calculated for resected nodules. Thyroid aspiration specimens interpreted as ND were excluded from these calculations^1,3^. Nodules with a benign FNA and final histology were considered true-negative cases. Surgically-confirmed malignant cases with FN/SFN, SM and malignant FNAs were regarded as true-positive results. AUS/FLUS nodules with malignant pathology on resection were also considered true-positive cases. However, since the typical primary management process for AUS/FLUS lesions is not surgical resection^4,5^, diagnostic indicators were re-assessed after excluding this category from analysis. These diagnostic values of inadequate samples were compared with those of adequate ones utilizing Pearson’s Chi-square test.

SPSS Software (Version 23, SPSS Inc., Chicago, IL, USA) was used for statistical analyses. This study was approved by the Institutional Review Board (IRB) of Samsung Medical Center, and performed in accordance with relevant guidelines and regulations. The IRB waived the requirement for informed consent because all patient data was de-identified.

## Electronic supplementary material

Supplemenatry Table S1-S3

## Data Availability

The data that support the findings of this study are available from the corresponding author upon reasonable request and with permission of Samsung Medical Center.
