# Supplementary material for: Modified Bethesda system informing cytopathologic adequacy improves malignancy risk stratification in nodules considered benign or atypia(follicular lesion) of undetermined significance
Source: Sci Rep. 2018 Sep 10;8:13503. doi: 10.1038/s41598-018-31955-9 (PMC6131141; doi:10.1038/s41598-018-31955-9)
Supplement: Supplementary file 1 — Supplemenatry Table S1-S3 [file 41598_2018_31955_MOESM1_ESM.docx]

**Supplementary Information**

**Article in *Scientific Reports***

**Modified Bethesda system informing cytopathologic adequacy improves malignancy risk stratification in nodules considered benign or atypia(follicular lesion) of undetermined significance**

You-Bin Lee, Ji-Ye Kim, Haeyon Cho, Soo Yeon Hahn, Jung Hee Shin, Seung-Eun Lee, Ji Eun Jun, Sun Wook Kim, Jae Hoon Chung, Tae Hyuk Kim, Young Lyun Oh

**Supplementary Table S1.** Baseline characteristics of study population and included nodules with inadequate or adequate fine needle aspirations.

|  | Inadequate FNAs^*^ | Adequate FNAs^†^ | *p*-value |
| --- | --- | --- | --- |
| Patient characteristics | | |  |
| Number | 1,423 | 1,359 |  |
| Age (years) | 52.5 ±211.9 | 50.1 ±012.4 | < 0.001 |
| Female [n (%)] | 1,058 (74.3%) | 1,041 (76.6%) | 0.178 |
| Nodule characteristics | | |  |
| Number | 1,450 | 1,446 |  |
| Nodule size (cm) | 1.20 (0.80-2.10) | — |  |
| Surgically resected [n (%)] | 213 (14.7%) | 345 (23.9%) | < 0.001 |

Abbreviations: FNA: fine-needle aspiration

Continuous variables with normal distributions were expressed as mean ± standard deviation, whereas continuous variables with non-normal distributions were expressed as median (interquartile range).

^*^ Cases were collected from April 2011 to March 2016.

^†^ Cases were collected from July to December 2013.

**Supplementary Table S2.** Summary of histological follow-up for nodules with inadequate and adequate fine needle aspirations

|  | Nodules with inadequate FNAs^*^ | Nodules with adequate FNAs^†^ |
| --- | --- | --- |
| Histologic subtype | Number of nodules (%) | Number of nodules (%) |
| Malignant | | |
| Total | 146 (100.0) | 289 (100.0) |
| Papillary thyroid carcinoma | 136 (93.2) | 286 (99.0) |
| Follicular thyroid carcinoma | 8 (5.5) | 2 (0.7) |
| Poorly differentiated thyroid carcinoma | 1 (0.7) | 0 (0.0) |
| Medullary thyroid carcinoma | 1 (0.7) | 1 (0.3) |
| Benign | | |
| Total | 67 (100.0) | 56 (100.0) |
| Benign follicular nodule | 46 (68.7) | 29 (51.8) |
| Follicular adenoma | 21 (31.3) | 26 (46.4) |
| Hyalinizing trabecular tumor | 0 (0.0) | 1 (1.8) |

Abbreviations: FNA: fine-needle aspiration

^*^ Cases were collected from April 2011 to March 2016.

^†^ Cases were collected from July to December 2013.

**Supplementary Table S3.** Summary of sonographic profiles for nodules with inadequate fine needle aspirations^*^

| ATA nodule sonographic patterns^†^ | Number of nodules (%) |
| --- | --- |
| Benign | 9 (0.6) |
| Very low suspicion | 67 (4.6) |
| Low suspicion | 603 (41.6) |
| Intermediate suspicion | 415 (28.6) |
| Not applicable^‡^ | 100 (6.9) |
| High suspicion | 256 (17.7) |
| Total | 1450 (100.0) |

Abbreviations: ATA: American Thyroid Association

^*^ Cases were collected from April 2011 to March 2016.

^†^ Categorized according to recommendations of the 2015 revised American Thyroid Association (ATA) guidelines

^‡^ Isoechoic or hyperechoic solid nodule, or partially cystic nodule with eccentric solid areas, with microcalcification, irregular margin or extrathyroidal extension, or non-parallel orientation
